# Moderators of Effects of Internet-Delivered Exercise and Pain Coping Skills Training for People With Knee Osteoarthritis: Exploratory Analysis of the IMPACT Randomized Controlled Trial

**DOI:** 10.2196/10021

**Published:** 2018-05-09

**Authors:** Belinda J Lawford, Rana S Hinman, Jessica Kasza, Rachel Nelligan, Francis Keefe, Christine Rini, Kim L Bennell

**Affiliations:** ^1^ Centre for Health, Exercise and Sports Medicine Department of Physiotherapy, School of Health Sciences The University of Melbourne Melbourne Australia; ^2^ Department of Epidemiology and Preventive Medicine Monash University Melbourne Australia; ^3^ Duke Pain Prevention and Treatment Research Program Durham, NC United States; ^4^ John Theurer Cancer Center Department of Biomedical Research Hackensack University Medical Center Hackensack, NJ United States

**Keywords:** telerehabilitation, moderators, osteoarthritis, exercise

## Abstract

**Background:**

Internet-delivered exercise, education, and pain coping skills training is effective for people with knee osteoarthritis, yet it is not clear whether this treatment is better suited to particular subgroups of patients.

**Objective:**

The aim was to explore demographic and clinical moderators of the effect of an internet-delivered intervention on changes in pain and physical function in people with knee osteoarthritis.

**Methods:**

Exploratory analysis of data from 148 people with knee osteoarthritis who participated in a randomized controlled trial comparing internet-delivered exercise, education, and pain coping skills training to internet-delivered education alone. Primary outcomes were changes in knee pain while walking (11-point Numerical Rating Scale) and physical function (Western Ontario and McMaster Universities Osteoarthritis Index function subscale) at 3 and 9 months. Separate regression models were fit with moderator variables (age, gender, expectations of outcomes, self-efficacy [pain], education, employment status, pain catastrophizing, body mass index) and study group as covariates, including an interaction between the two.

**Results:**

Participants in the intervention group who were currently employed had significantly greater reductions in pain at 3 months than similar participants in the control group (between-group difference: mean 2.38, 95% CI 1.52-3.23 Numerical Rating Scale units; interaction *P*=.02). Additionally, within the intervention group, pain at 3 months reduced by mean 0.53 (95% CI 0.28-0.78) Numerical Rating Scale units per unit increase in baseline self-efficacy for managing pain compared to mean 0.11 Numerical Rating Scale units (95% CI –0.13 to 0.35; interaction *P*=.02) for the control group.

**Conclusions:**

People who were employed and had higher self-efficacy at baseline were more likely to experience greater improvements in pain at 3 months after an internet-delivered exercise, education, and pain coping skills training program. There was no evidence of a difference in the effect across gender, educational level, expectation of treatment outcome, or across age, body mass index, or tendency to catastrophize pain. Findings support the effectiveness of internet-delivered care for a wide range of people with knee osteoarthritis, but future confirmatory research is needed.

**Trial Registration:**

Australian New Zealand Clinical Trials Registry ACTRN12614000243617; https://www.anzctr.org.au/Trial/Registration/TrialReview.aspx?id=365812&isReview=true (Archived by WebCite at http://www.webcitation.org/6z466oTPs)

## Introduction

Knee osteoarthritis (OA) is prevalent, affecting approximately one-quarter of adults [1]. People with knee OA often experience persistent pain, impaired function, reduced quality of life, and psychological disability [2]. Education and exercise are key nonsurgical, nondrug strategies recommended by all clinical guidelines for the management of knee OA [3]. In addition, because low self-efficacy, increased pain catastrophizing, and maladaptive pain coping strategies are common among people with OA [4-6], interventions that target these psychological factors may also provide benefits. Growing evidence supports the use of pain coping skills training (PCST) to improve pain and physical and psychological functioning in chronic pain conditions [7-9]. However, many people with OA may have difficulties accessing health professionals skilled in the prescription of exercise or psychological interventions [10,11]. As such, more accessible models of health service delivery are needed.

Providing care remotely via the internet is one way to potentially improve access to treatments for people with OA. For example, programs such as Skype or FaceTime are freely available, offering potentially accessible ways in which people can consult via video with health professionals such as physical therapists. Our recent randomized controlled trial (RCT) found that an internet-delivered intervention combining online educational material, an automated PCST program, and physical therapist-prescribed exercise via Skype led to greater improvements in pain and function in people with knee OA compared to online educational material alone [12]. These improvements were also maintained at long-term follow-up (9 months). These findings are consistent with evidence in other populations, where care delivered via videoconferencing for people who have undergone knee joint replacement surgery led to improvements in physical activity and function that were similar to those after face-to-face consultations [13]. Collectively, these studies provide support for the effectiveness of telerehabilitation as an alternative model of service delivery.

When evaluating novel models of service delivery, it is important to consider whether there are subgroups of patients that respond more or less favorably. Moderation analysis of RCT data can be used to determine if the effect of a treatment relative to the control differs for distinct subgroups of patients. In these analyses, baseline characteristics that interact with the treatment to influence clinical outcomes are identified as moderators [14]. As such, the aim of moderation analysis is to determine whether groups of people with different characteristics respond differently to the treatment. Identifying moderators of the effect of a treatment on an outcome can help match patients to the most effective and appropriate treatment thereby improving outcomes, in turn helping to control treatment costs and promoting efficient use of resources. In addition, moderation analyses can help guide future refinements to an intervention so that it may be more effective for subgroups who do not respond as well.

To our knowledge, few previous studies have conducted moderator analyses on outcomes following an intervention involving exercise or PCST for people with OA. There is some evidence that pain coping style, expectation of treatment response, radiographic disease severity, age, and educational level may moderate the effects of a face-to-face PCST intervention on treatment outcomes among people with hip and knee OA [15]. Another study found that body mass index (BMI) moderated the effects of a supervised aquatic exercise intervention on changes in quality of life among people with OA [16]. Although a number of studies have identified predictors of outcomes following exercise for people with OA (eg, self-efficacy for managing pain [17], gender [18], and age [19]), outcome predictors are not necessarily the same as moderators of the effect of a treatment. For example, participants with a particular characteristic may improve over time no matter what treatment they receive and, although that characteristic might predict improvement, it may not necessarily moderate the effect of a treatment. Thus, predictors of outcome do not allow identification of subgroups that respond, or do not respond, to a given intervention. Therefore, the aim of this study was to explore potential demographic and clinical moderators of the effect of a combined internet-delivered exercise, education, and PCST program on changes in pain and physical function for people with knee OA. This will provide future directions for confirmatory studies.

## Methods

This study involved exploratory moderation analyses using data from a parallel, two-group pragmatic RCT (Australian New Zealand Clinical Trials Registry: ACTRN12614000243617) aiming to evaluate the effectiveness of internet-delivered physical therapist-prescribed home exercise, education, and PCST compared to internet-delivered education alone (IMPACT trial). Study procedures were approved from the University of Melbourne Human Research Ethics Committee and all participants provided written informed consent. The trial protocol [20] and outcomes [12] have been published.

### Study Population

A total of 148 people with chronic knee pain were recruited Australia-wide to participate in the RCT. Briefly, inclusion criteria included age 50 years or older, knee pain for more than 3 months and on most days of the previous month, knee pain during walking in the previous week (≥4 on an 11-point Numerical Rating Scale [NRS]), mild to moderate physical dysfunction (>20 out of 68 on the physical function subscale of the Western Ontario and McMaster Universities Osteoarthritis Index [WOMAC]), and having an active email account and computer with internet access.

### Intervention

Participants in the intervention arm of the trial received three internet-delivered treatments: (1) educational material about exercise and physical activity, pain management, emotions, healthy eating, complementary therapies, and medications (freely available on the Arthritis Australia website [21]); (2) an online interactive automated PCST program [9,22], involving completion of one 35 to 45 minute training module per week for 8 weeks; and (3) seven physical therapist consultations via Skype over 12 weeks, with each consultation lasting 30 to 45 minutes. Participants were randomly allocated to one of eight different physical therapists, who delivered all subsequent Skype sessions. Physical therapists performed a brief assessment and prescribed a lower-limb strengthening home exercise program to be completed by the participant three times per week. Participants were also encouraged to increase their physical activity levels and were given the opportunity to use a pedometer for motivation if desired, and were also encouraged to practice pain coping skills daily.

The control group received access to the same educational material as the intervention group (ie, material about exercise and physical activity, pain management, emotions, healthy eating, complementary therapies, and medications through the Arthritis Australia website), but did not have access to the PCST program.

### Dependent Variables

Participants completed questionnaires online at baseline, 3 months, and 9 months. Primary outcomes were valid and reliable measures of pain and function [23]. Pain during walking over the last week was measured using an 11-point NRS ranging from 0 (“no pain”) to 10 (“worst pain possible”). Function was measured using the WOMAC physical function subscale [24] with scores ranging from 0 (no dysfunction) to 68 (maximum dysfunction). Thus, we calculated the 3-month change in pain and function as baseline minus 3-month values, and 9-month change in pain and function as baseline minus 9-month values.

### Selected Moderators

Based on previous research [15-19] and/or theoretical plausibility (Multimedia Appendix 1), we investigated whether the effect of the internet-delivered treatment relative to the control treatment was moderated by each of the following baseline variables: gender, age, level of education (dichotomized as no tertiary training or some tertiary training), employment situation (dichotomized as employed or not employed), pain self-efficacy using the pain subscale of the Arthritis Self-Efficacy Scale [25] (scores ranging from 1 to 10, with higher scores indicating greater self-efficacy), pain catastrophizing using the Pain Catastrophizing Scale [26] (scores ranging from 0 to 52, with higher scores indicating greater catastrophizing), expectation of treatment effects using a five-point Likert scale ranging from “no effect” to “complete recovery” (dichotomized into “no effect to moderate improvement” or “large improvement to complete recovery”), and BMI based on self-reported height and weight.

### Analysis

To determine whether the effect of the treatment relative to the control on change in each of the primary outcomes was moderated by the selected baseline variables, separate linear regression models were fit with the potential moderator variable and study group as covariates, and an interaction between the two. This analysis provides an answer to the question of whether the effect of the treatment, relative to the control condition, differs for different subgroups of participants. For binary moderators, the estimated effect of treatment and a 95% confidence interval was determined for each of the moderator levels. For continuous moderators, results were calculated as the effect of a one-unit increase of that moderator in each of the control and intervention groups. The linear regression assumptions of linearity, heteroscedasticity, and normality were assessed using standard diagnostic plots. Scatterplots of the outcome against each continuous moderator by treatment group were examined to determine if more complex models (including nonlinear terms for moderators) were warranted. All statistical analyses were performed using Stata version 14.1 (StataCorp LLC, College Station, TX, USA).

## Results

A total of 148 people with knee OA were enrolled in the study, with just over half female (56.1%, 83/148) and currently employed either full-time or part-time (57.4%, 85/148; Table 1). At baseline, participants in the intervention group had higher educational levels than those in the control group. At 9 months, nine people in the intervention group and seven people in the control group had been lost to follow-up (unable to contact, family issues/illness, deceased).

### Moderators of the Effect of the Intervention on Change in Walking Pain

There was no evidence for moderation of the effect of the intervention on change in walking pain at 3 or 9 months by most binary variables, including gender, level of education, and treatment expectations (Table 2). There was some evidence for moderation of the treatment effect by employment status for change in walking pain at 3 months (interaction *P*=.02). Among those who were currently employed, participants assigned to the intervention group had greater reductions in pain than those in the control group, with an estimated difference between groups of mean 2.38 (95% CI 1.52-3.23) NRS units. Among unemployed participants, the estimated difference in reduction in pain at 3 months between the intervention and control groups was mean 0.86 (–0.13 to 1.85) NRS units.

**Table 1:** Baseline descriptive characteristics (N=148).

Characteristic		Intervention (n=74)	Control (n=74)
Age (years), mean (SD)		60.8 (6.5)	61.5 (7.6)
Body mass index (kg/m^2^), mean (SD)		32.0 (13.9)	30.1 (10.2)
Gender (female), n (%)		43 (58)	40 (54)
**Level of education, n (%)**			
	No tertiary training	16 (22)	24 (32)
	Some tertiary training	58 (78)	50 (68)
**Employment status, n (%)**			
	Employed	40 (54)	45 (61)
	Unemployed	34 (46)	29 (39)
**Expectation of treatment outcomes, n (%)**			
	No effect to moderate improvement	11 (15)	21 (29)
	Large improvement to complete recovery	63 (85)	52 (71)
Self-efficacy (pain)^a^		6.1 (1.8)	5.9 (1.8)
Pain catastrophizing^b^		8.8 (9.2)	10.1 (9.6)

^a^ASES: Arthritis Self-Efficacy Scale (range 1-10; higher scores indicate greater self-efficacy).

^b^PCS: Pain Catastrophizing Scale (range 0-52; higher scores indicate greater catastrophizing).

**Figure 1:**
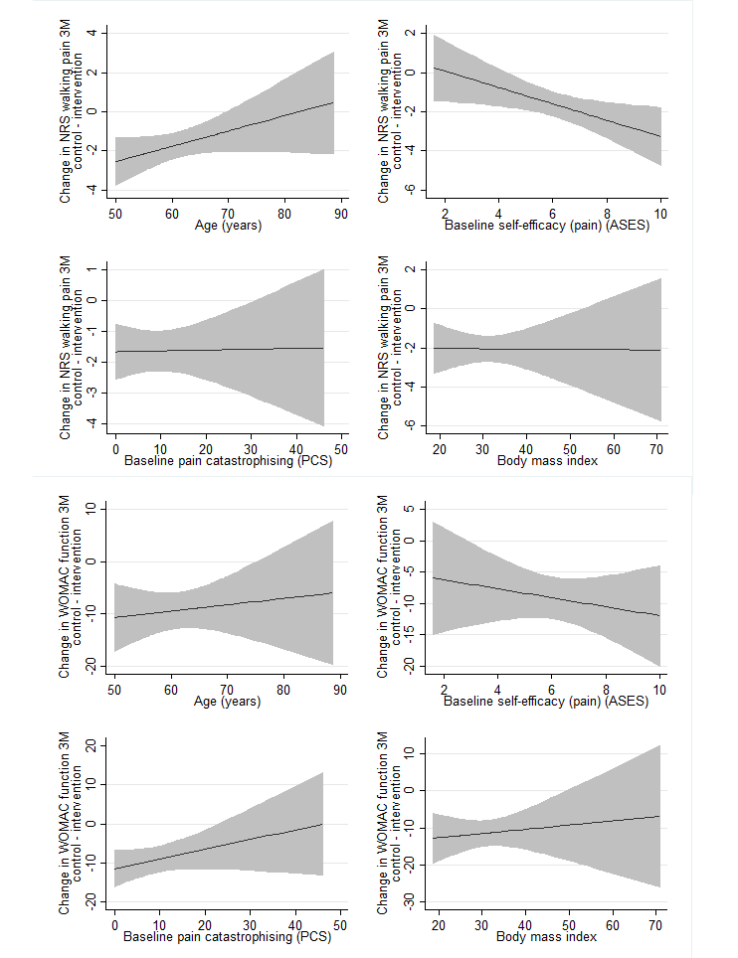
Difference in change in NRS walking pain and WOMAC function (baseline-3 months) between treatment groups for each potential continuous moderator. Negative values favor the intervention group. Solid line indicates the difference between the control and intervention arms. Shaded areas indicate 95% confidence intervals. ASES: Arthritis Self-Efficacy Scale (range 1-10; higher scores indicate greater self-efficacy); NRS: Numerical Rating Scale (range 0-10; lower scores indicate less pain); PCS: Pain Catastrophizing Scale (range 0 to 52; higher scores indicate greater catastrophizing); WOMAC: Western Ontario and McMaster Universities Osteoarthritis Index; ranges from 0 to 68, where lower scores indicate better function.

**Figure 2:**
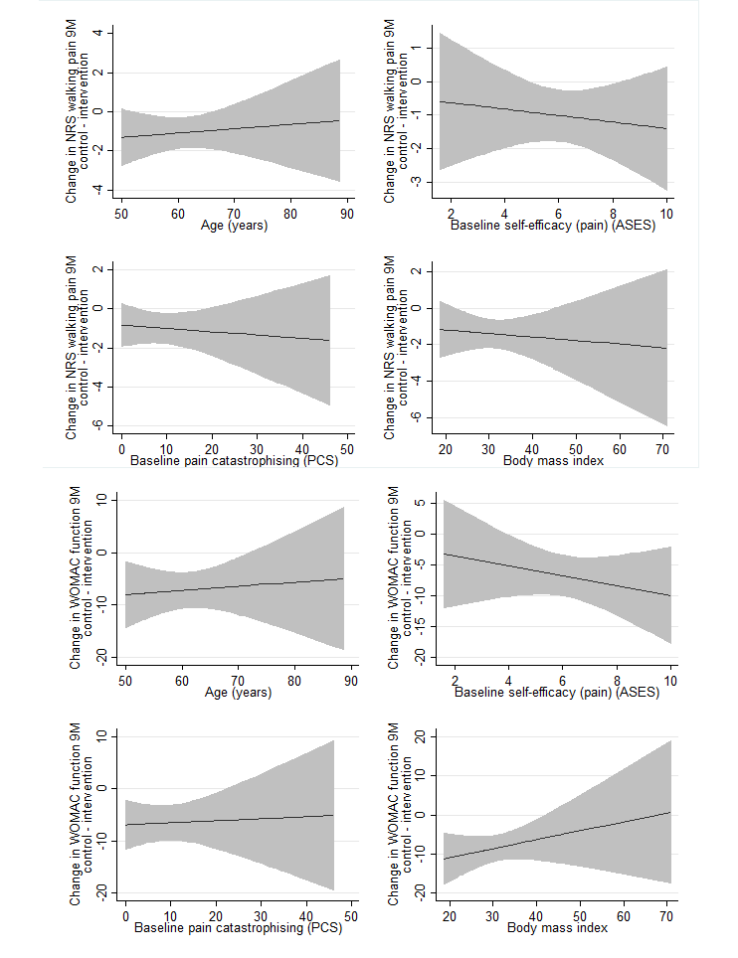
Difference in change in NRS walking pain and WOMAC function (baseline–9 months) between treatment groups for each potential continuous moderator. Negative values favor the intervention group. Solid line indicates the difference between the control and intervention arms. Shaded areas indicate 95% confidence intervals. ASES: Arthritis Self-Efficacy Scale (range 1-10; higher scores indicate greater self-efficacy); PCS: Pain Catastrophizing Scale (range 0-52; higher scores indicate greater catastrophizing); WOMAC: Western Ontario and McMaster Universities Osteoarthritis Index (range 0-68, lower scores indicate better function).

Scatterplots of changes in pain against each continuous moderator (Multimedia Appendices 2 and 3) indicated that exploration of linear terms for each moderator was sufficient, and more complex models were not warranted. Results of linear models for each of the continuous moderators are presented in Figures 1 and 2. There was no evidence that age, pain catastrophizing, and BMI interacted with treatment group in the model for changes in walking pain at 3 or 9 months (Table 3). However, there was evidence that self-efficacy for managing pain interacted with treatment group at 3 months (*P*=.02). Within the intervention group, for each additional unit of self-efficacy (pain) at baseline, pain at 3 months reduced by an additional mean 0.53 (95% CI 0.28-0.78) NRS units, whereas in the control group, the association between self-efficacy (pain) and change in pain was estimated as mean 0.11 (95% CI –0.13 to 0.35) NRS units. That is, people with higher self-efficacy for managing pain at baseline demonstrated greater reductions in walking pain with the intervention compared to control.

### Moderators of the Effect of the Intervention on Change in Physical Function

Scatterplots of changes in function against each continuous moderator (Multimedia Appendices 2 and 3) indicated that exploration of linear terms for each moderator was sufficient. Results of linear models are presented in Figures 1 and 2. None of the selected binary (Table 2) or continuous (Table 3) moderators had a significant interaction with the treatment in the model for changes in physical function at 3 or 9 months.

**Table 2:** Results of the moderation analysis for binary moderators for change in walking pain and physical function.

Moderator			3 months				9 months			
			Intervention-control difference (95% CI)^a^		Interaction (*P* value)		Intervention-control difference (95% CI)^b^		Interaction (*P* value)	
**Change in walking pain**										
	**Gender**				.64				.45	
		Male		1.88 (0.85, 2.90)				0.74 (–0.47, 1.94)		
		Female		1.56 (0.69, 2.42)				1.33 (0.31, 2.36)		
	**Level of education**				.22				.58	
		No tertiary training		0.96 (–0.29, 2.21)				0.57 (–0.97, 2.12)		
		Some tertiary training		1.87 (1.10, 2.64)				1.08 (0.18, 1.97)		
	**Treatment expectation**				.70				.85	
		No effect-moderate improvement		1.76 (1.01, 2.51)				1.08 (0.22, 1.94)		
		Large improvement/complete recovery		1.45 (0.06, 2.85)				1.26 (-0.41, 2.93)		
	**Employment**				.02				.86	
		Not employed		0.86 (–0.13, 1.85)				1.06 (–0.13, 2.25)		
		Employed		2.38 (1.52, 3.23)				1.20 (0.17, 2.22)		
**Change in physical function**										
	**Gender**				.43				.88	
		Male		7.80 (2.46, 13.14)				6.87 (1.63, 12.12)		
		Female		10.63 (6.11, 15.15)				7.42 (2.94, 11.90)		
	**Level of education**				.22				.25	
		No tertiary training		5.66 (–0.88, 12.20)				3.25 (–3.46, 9.96)		
		Some tertiary training		10.44 (6.41, 14.48)				7.75 (3.86, 11.64)		
	**Treatment expectation**				.73				.99	
		No effect-moderate improvement		9.77 (5.87, 13.69)				7.25 (3.46, 11.04)		
		Large improvement/complete recovery		8.35 (1.05, 15.65)				7.27 (–0.12, 14.65)		
	**Employment**				.14				.81	
		Not employed		6.88 (1.74, 12.01)				6.72 (1.50, 11.93)		
		Employed		11.94 (7.48, 16.41)				7.57 (3.05, 12.08)		

^a^Baseline-3 months.

^b^Baseline-9 months.

**Table 3:** Results of the moderation analysis for continuous moderators for change in walking pain and physical function.

Moderator		3 months						9 months					
		Estimated moderator coefficient (95% CI)				Interaction (*P* value)		Estimated moderator coefficient (95% CI)				Interaction (*P* value)	
		Control group		Intervention group				Control group		Intervention group			
**Change in walking pain**													
	Age (years)		0.02 (–0.04, 0.08)		–0.06 (–0.13, 0.01)		.10		–0.01 (–0.08, 0.06)		–0.03 (–0.12, 0.05)		.69
	Self-efficacy (pain)^a^		0.11 (–0.13, 0.35)		0.53 (0.28, 0.78)		.02		0.21 (–0.08, 0.51)		0.31 (0.00, 0.61)		.66
	Pain catastrophizing^b^		–0.04 (–0.09, 0.01)		–0.04 (–0.09, 0.01)		.94		0.01 (–0.01, 0.03)		0.02 (–0.00, 0.04)		.49
	Body mass index		–0.02 (–0.09, 0.04)		–0.02 (–0.09, 0.04)		.98		–0.07 (–0.15, 0.01)		–0.05 (–0.12, 0.02)		.72
**Change in physical function**													
	Age (years)		–0.08 (–0.40, 0.23)		–0.21 (–0.58, 0.17)		.62		–0.01 (–0.32, 0.31)		–0.09 (–0.46, 0.29)		.74
	Self-efficacy (pain)^a^		0.96 (–0.35, 2.26)		1.67 (0.32, 3.03)		.45		0.88 (–0.40, 2.17)		1.68 (0.36, 3.00)		.39
	Pain catastrophizing^b^		–0.14 (–0.39, 0.11)		–0.39 (–0.64,–0.13)		.17		–0.03 (–0.13, 0.07)		0.04 (–0.05, 0.14)		.28
	Body mass index		–0.08 (–0.44, 0.28)		–0.19 (–0.52, 0.14)		.64		–0.04 (–0.38, 0.30)		–0.27 (–0.59, 0.05)		.32

^a^ASES: Arthritis Self-Efficacy Scale (range 1-10; higher scores indicate greater self-efficacy).

^b^PCS: Pain Catastrophizing Scale (range 0-52; higher scores indicate greater catastrophizing).

## Discussion

### Principal Findings

The aim of this study was to explore potential demographic and clinical moderators of the effect of an internet-delivered exercise, education, and PCST program for people with knee OA on changes in pain and physical function. We found that the effect of the intervention at 3 months differed by employment status and self-efficacy, such that the effect of the intervention on reductions in pain was greater among those who were employed and had higher self-efficacy for managing their pain at baseline. There was no evidence that any of the selected baseline variables moderated the effect of the intervention on changes in physical function at 3 months, and no evidence that any moderated the effect of the intervention on changes in pain or function at 9 months.

We found some evidence that employment status moderated the effect of the intervention on changes in walking pain at 3 months. Those who were currently employed had greater improvements in walking pain with the treatment compared to the control, whereas among those who were not employed there was no difference in changes on walking pain at 3 months between the intervention and control groups. Wright and colleagues [19] found that employment status did not significantly predict changes in pain or function after an exercise and/or manual therapy intervention for people with hip OA. However, they looked at predictors, rather than moderators, and used seven different categories to define employment status (ranging from “homemaker” to “full-time or part-time employment”) with a small number of participants in each group, which may have limited the study’s power. Being employed exposes people to a range of psychosocial environments and experiences (eg, structured and meaningful use of time, opportunities to use new and existing skills, variety in tasks, and social contact outside of the home) that have been linked to decreases in emotional distress within chronic pain populations, independent of pain severity [27-29]. People who are employed may also be more motivated to improve so that they can continue working; furthermore, being employed has been associated with higher self-efficacy among people with chronic musculoskeletal pain [30,31], which may have contributed to greater reduction in pain with treatment at 3 months. However, there was no evidence that employment status moderated treatment effects on changes in pain at 9 months. The reasons for this are not clear, but support the longer-term effectiveness of this intervention for people who are employed or unemployed.

To our knowledge, no previous research has investigated self-efficacy as a potential moderator of an exercise or PCST intervention on outcomes in people with chronic pain conditions. Skou and colleagues [17] found that self-efficacy for managing pain at 3 months predicted pain and quality-of-life outcomes at 1 year among people with knee or hip OA who received an education and exercise intervention. However, they did not investigate whether baseline self-efficacy also predicted outcomes and only looked at predictors of outcomes, rather than moderators of the effect of treatment. According to Social Cognitive Theory [32], self-efficacy influences a person’s choice of effort and persistence in the face of adversity (eg, knee pain) [33]. As such, our findings make intuitive sense in that people who are more confident in their ability to succeed despite the presence of pain are more likely to experience greater improvements with a treatment program that emphasizes self-management in the form of home exercise and utilizing pain coping skills. However, there was no evidence that self-efficacy for managing pain moderated intervention effects on changes in pain at 9 months. Although the reasons for this are not clear, the results support the longer-term effectiveness of the intervention for people with either high or low self-efficacy at baseline.

In contrast to previous studies exploring moderators of PCST [15] and exercise [16], we found no evidence that age, BMI, expectation of treatment outcome, pain coping style, and education moderated the effect of the intervention relative to the control on changes in pain and function. In the literature, there appears to be some heterogeneity in moderators of treatment effects following exercise or PCST interventions for people with chronic pain conditions [34], which might be because of differences in intervention design or delivery across studies. Our intervention was unique in that it was remotely delivered and combined both physical and psychological interventions. Other studies involved face-to-face supervised exercise programs [16,35] or 10 sessions of PCST delivered face-to-face by nurse practitioners [15]. The exercise interventions of other studies also differed to ours, in which they investigated moderators of aquatic exercise for people with OA [16] or a combined intervention of dietary weight loss and exercise (combining aerobic and resistance training) [35]. In addition, our sample comprised fewer females who were also, on average, younger than those of previous studies [15,16,35]. These differences in delivery mode and frequency/type of exercise/PCST, as well as heterogeneity in sample characteristics and measures of outcomes or moderator variables, might explain the differences between our findings and the limited existing literature.

To our knowledge, this is the first study to explore moderators of the effect of internet-delivered care on outcomes for people with a chronic musculoskeletal condition. We found no evidence that age or level of education moderated the effect of internet-delivered care on changes in pain or function at 3 or 9 months. Similarly, previous studies found that age had no association with outcomes following an intervention involving behavioral treatment delivered via Skype for veterans with posttraumatic stress disorder [36,37]. Our recent survey also found that, among people with knee and/or hip OA, there was no evidence that age and level of education influenced interest in receiving exercise from a physical therapist via video [38]. These findings counter commonly held misconceptions of telehealth, namely that it may be most suitable for people who are younger and/or highly educated, and are therefore “technology savvy” [39,40]. Our findings suggest that people of varying ages, and those with high or low levels of education, benefited just as much with internet-delivered care. This study thus provides further support for an increased use of telerehabilitation as an alternative model of service delivery for people with OA.

Our findings have a number of practical implications. Internet-delivered education, exercise, and PCST appears to be effective for a range of people with OA, including both men and women with varying educational backgrounds, of different ages, as well as those who are obese or normal weight. This suggests that the intervention does not need to be targeted at specific subgroups of patients. For greater improvements in pain at 3 months, or if services or resources are limited, service providers or physical therapists could consider using measures to screen for people with low self-efficacy for managing pain as a means of identifying potentially vulnerable patients who might need some additional support before receiving this kind of intervention or an alternative such as face-to-face care.

### Strengths and Limitations

This study has a number of strengths, including the robust study design (moderation/subgroup analysis using RCT data), the range of potential moderators included, the use of meaningful patient-reported clinical outcomes of pain and physical function, and the fact that we recruited participants from regional/remote and metropolitan areas across Australia. This study also has some limitations. Because the analyses in our study were exploratory in nature, our results should be interpreted with caution. For example, it is possible that some of the interactions in our analysis were nonsignificant due to lack of power rather than the absence of a significant effect. As such, further research is required to confirm our findings. Our intervention included multiple components (ie, Skype-delivered exercise in addition to PCST and online educational materials), so it is not possible to determine which treatment component interacts with each of the moderator variables. In addition, more than 75% of the people in the intervention arm of the study had completed some tertiary education and, therefore, our results may not be generalizable to people who are less educated.

### Conclusions

In summary, people who were employed and had higher self-efficacy at baseline were more likely to experience greater improvements in pain at 3 months after an internet-delivered exercise, education, and PCST program. This may be because people who are more confident in their ability to succeed despite the presence of pain are more likely to experience greater improvements with a treatment program that emphasizes self-management. There was no evidence of a difference in the effect across gender, educational level, expectation of treatment outcome, or across age, BMI, or tendency to catastrophize pain. This study provides further support for telerehabilitation as an alternative model of service delivery that is suitable for a broad range of people with OA. Further research is required to confirm our findings and identify moderators of the effect of exercise and PCST on long-term changes in pain and function, and also identify moderators of the effects of other modes of service delivery (eg, telephone-delivered care).

## Appendix

Multimedia Appendix 1 Overview of selected demographic and clinical moderators.

Multimedia Appendix 2 Change in NRS walking pain and WOMAC function (baseline-3 months) against each continuous potential moderator, by treatment group.

Multimedia Appendix 3 Change in NRS walking pain and WOMAC function (baseline-9 months) against each continuous potential moderator, by treatment group.

## Abbreviations

ASES: Arthritis Self-Efficacy Scale
BMI: body mass index
NRS: Numerical Rating Scale
OA: osteoarthritis
PCST: pain coping skills training
RCT: randomized controlled trial
WOMAC: Western Ontario and McMaster Universities Osteoarthritis Index
