# Supplementary material for: Moderators of Effects of Internet-Delivered Exercise and Pain Coping Skills Training for People With Knee Osteoarthritis: Exploratory Analysis of the IMPACT Randomized Controlled Trial
Source: J Med Internet Res. 2018 May 9;20(5):e10021. doi: 10.2196/10021 (PMC5966648; doi:10.2196/10021)
Supplement: Multimedia Appendix 1 [file jmir_v20i5e10021_app1.pdf]

Appendix

Table 1. Overview of selected demographic and clinical moderators

Selected moderator variables	Justification
Age<br>Level of education<br>Expectation of treatment effects	Older age, higher education, higher expectations associated with better outcomes in pain, quality of life, pain catastrophising, self-efficacy, and fatigue after face-to-face pain coping skills training [1]
Gender	Being male associated with better outcomes in pain and physical function after supervised strengthening exercises [2]
Pain self-efficacy	Higher self-efficacy associated with better outcomes in pain and quality of life after supervised neuromuscular exercise [3]
BMI	Being obese associated with better outcomes in quality of life after supervised aquatic exercise [4]
Employment situation	Chosen based on theoretical plausibility. An internet delivered intervention could be perceived as being more convenient, flexible and hence more effective by those who are employed than by those who are not employed.
Pain catastrophising	Chosen based on theoretical plausibility and indirect evidence whereby pain catastrophizing is related to pain severity, psychological and physical disability, walking speed, and poor outcomes after pain treatment [5,6]. People with lower pain catastrophising may be more likely to engage with, and adhere to, a self-management intervention.

BMI: body mass index

1. Broderick, J.E., F.J. Keefe, S. Schneider, D.U. Junghaenel, P. Bruckenthal, J.E. Schwartz, A.T. Kaell, D.S. Caldwell, D. McKee, and E. Gould, Cognitive behavioral therapy for chronic pain is effective, but for whom? Pain, 2016. 157(9): p. 2115-2123.
2. French, H.P., R. Galvin, T. Cusack, and G.M. McCarthy, Predictors of short-term outcome to exercise and manual therapy for people with hip osteoarthritis. Physical Therapy, 2014. 94(1): p. 31.
3. Skou, S.T., M.E. Simonsen, A. Odgaard, and E.M. Roos, Predictors of long-term effect from education and exercise in patients with knee and hip pain. Danish Medical Journal, 2014. 61(7): p. A4867.
4. Cadmus, L., M.B. Patrick, M.L. Maciejewski, T. Topolski, B. Belza, and D.L. Patrick, Community-based aquatic exercise and quality of life in persons with osteoarthritis. Medicine & Science in Sports & Exercise, 2010. 42(1): p. 8-15.
5. Somers, T.J., F.J. Keefe, J.J. Pells, K.E. Dixon, S.J. Waters, P.A. Riordan, J.A. Blumenthal, D.C. McKee, L. LaCaille, and J.M. Tucker, Pain catastrophizing and pain-related fear in osteoarthritis patients: relationships to pain and disability. Journal of Pain and Symptom Management, 2009. 37(5): p. 863-872.
6. Edwards, R.R., C.O. Bingham, J. Bathon, and J.A. Haythornthwaite, Catastrophizing and pain in arthritis, fibromyalgia, and other rheumatic diseases. Arthritis Care & Research, 2006. 55(2): p. 325-332.
