# Supplementary figures and images for: Moderators of Effects of Internet-Delivered Exercise and Pain Coping Skills Training for People With Knee Osteoarthritis: Exploratory Analysis of the IMPACT Randomized Controlled Trial
Source: J Med Internet Res. 2018 May 9;20(5):e10021. doi: 10.2196/10021 (PMC5966648; doi:10.2196/10021)

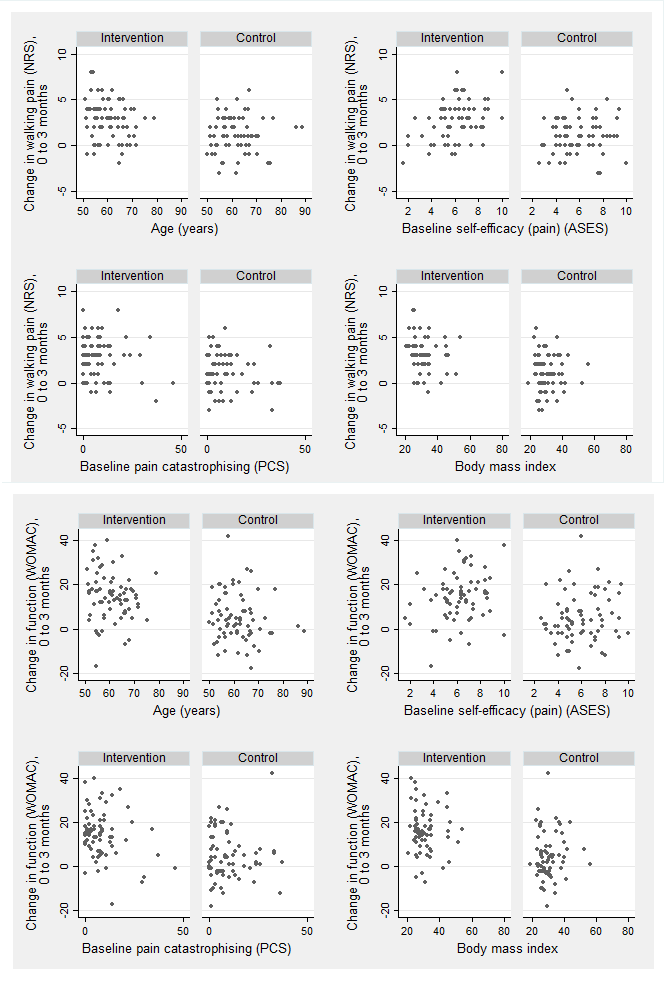

Supplement: Multimedia Appendix 2 [file jmir_v20i5e10021_app2.png]

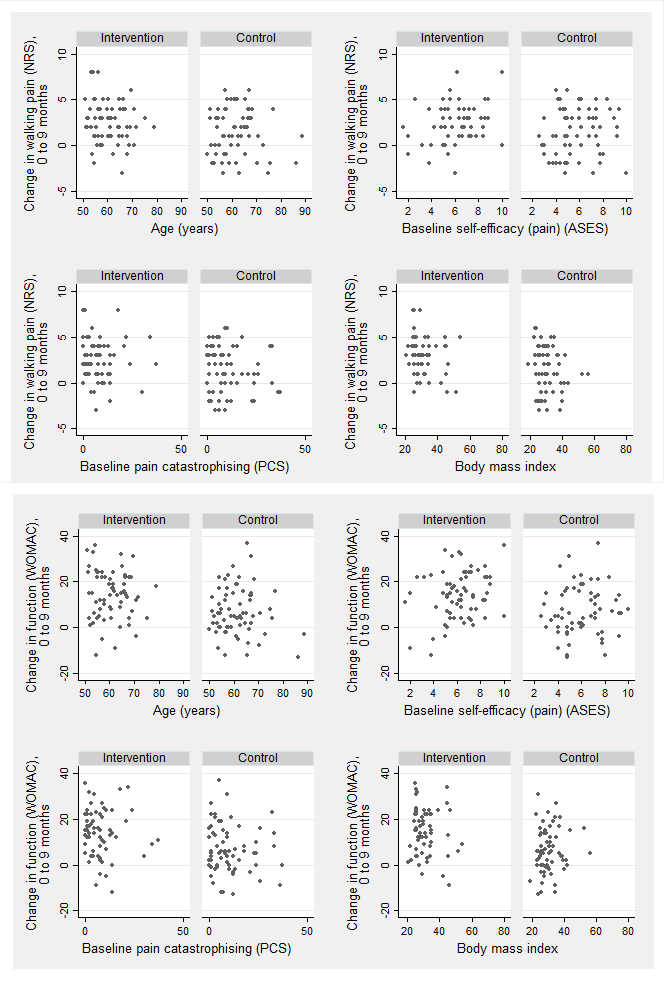

Supplement: Multimedia Appendix 3 [file jmir_v20i5e10021_app3.png]
